# Distal lordotic angle independent of pelvic incidence to reduce the risk of adjacent segment degeneration following L4–S1 posterior lumbar interbody fusion: A retrospective study

**DOI:** 10.1097/MD.0000000000047329

**Published:** 2026-01-23

**Authors:** Young-Gook Gwak, Myung-Hoon Shin

**Affiliations:** a Department of Neurosurgery, Incheon St Mary’s Hospital, College of Medicine, The Catholic University, Incheon, Republic of Korea.

**Keywords:** adjacent segment degeneration, distal lumbar lordosis, lumbar lordosis distribution, posterior lumbar interbody fusion, proximal lumbar lordosis

## Abstract

Pelvic incidence (PI)-based lumbar lordosis (LL) restoration is crucial for spinal balance. Proper proximal and distal LL distribution reduces mechanical complications, but the effect of segmental lordosis at L4–S1 on adjacent segment degeneration (ASD) remains unclear. This study aims to determine whether the lordotic angle achieved during L4–5–S1 posterior lumbar interbody fusion (PLIF) is associated with the incidence of postoperative ASD. This retrospective study analyzed radiographic data from L4–5–S1 PLIF patients (January 2019–December 2021) with at least 2 years of follow-up. Patients were categorized into ASD and non-ASD groups. Radiographic parameters, including PI, LL, upper lumbar lordosis, lower lumbar lordosis, and the lordosis distribution index, were compared. Correlation and stratification analyses were performed. A total of 155 patients were included (84 non-ASD, 71 ASD). The mean follow-up was 39.60 ± 20.33 months, with a 7.7% revision rate. The ASD group had a significant PI–LL mismatch (19.48 ± 11.56° vs 9.98 ± 10.07°, *P* < .001) and a lower L4–S1 lordosis angle (24.73 ± 10.86° vs 30.93 ± 6.97°, *P* = .005). In the non-ASD group, the L1–L4 angle correlated positively with PI (*r* = 0.643, *P* < .001), but L4–S1 did not (*r* = −0.027, *P* = .806). In contrast, the ASD group showed a positive correlation between L4–S1 and PI (*r* = 0.409, *P* < .001). PI stratification revealed stable distal lordosis (L4–S1) in the non-ASD group (*P* = .758) but significant variation in the ASD group (*P* = .003). These observations led the authors to infer that the stability of distal segmental lordosis – particularly at L4–S1 – across varying PI values may be a distinguishing feature associated with lower ASD risk. Proximal lordosis varies with PI, whereas distal lordosis remains stable in non-ASD patients. Insufficient distal lordosis may contribute to ASD, highlighting the importance of optimizing LL distribution during L4–S1 PLIF.

## 1. Introduction

During its initial introduction, advocates of posterior lumbar interbody fusion (PLIF) claimed that the procedure was effective in preventing the collapse of the disc space and maintaining the neural foramen height, thus protecting against nerve root compression caused by bony compression.^[1]^ Since then, PLIF has garnered widespread acceptance for the treatment of various lumbar spinal pathologies, including lumbar disc herniation, lumbar spinal stenosis, spondylolisthesis, adult spinal deformity, and traumatic vertebral fractures. The increased popularity of PLIF is largely attributed to the introduction of pedicle screw instrumentation, which permits additional fixation, and the evolution of advanced graft materials that promote solid arthrodesis and favorable clinical outcomes.^[2,3]^ Although successful arthrodesis at the index level may lead to clinical improvement, it is believed to inevitably result in changes in the normal biomechanics of the spine, which in turn accelerates degenerative changes in the adjacent unfused segment.^[4]^ The potential risk factors of adjacent segment degeneration (ASD) comprised injury to the nearby facet joint, fusion length, sagittal alignment, preexisting degenerative discs adjacent to the affected area, spinal stenosis, age, osteoporosis, female gender, and postmenopausal state.^[5]^

It is widely acknowledged that a significant relationship exists between pelvic parameters and lumbar curvature as one moves from the pelvis to the lumbar region.^[6]^ The SRS-Schwab classification^[7]^ proposed clinical guidelines for appropriate lumbar lordosis (LL) based on its strong correlation with pelvic incidence (PI). The recommended range for LL is within 10° of PI, and the pelvic tilt (PT) should be <20°. Various studies^[7–10]^ have reported that achieving these specific targets can effectively prevent ASD and improve patients’ quality of life after lumbar fusion surgery. Not only is the overall degree of lordosis related to PI, it is also imperative to achieve an optimal distribution of lordosis between the proximal (L1–L4) and distal components (L4–S1) to prevent the onset of ASD.^[11,12]^ Roussouly et al^[13]^ offered insights into the conceptual basis of the lordosis distribution index (LDI) by expounding upon the normal variation in sagittal plane alignment at the lumbosacral junction. LDI is a radiographic parameter that assesses the ratio of lordosis originating from the distal lumbar segment. According to their findings, as PI values increased, more vertebral bodies were recruited, resulting in lower LDI values. Yilgor et al^[14]^ proposed a PI-specific global alignment and proportion score to predict the likelihood of mechanical complications following lumbar fusion surgery. They suggested that optimal alignment could be achieved when LDI values were restricted to the range of 50%–80%.

Upon conducting a comprehensive analysis, Pesenti et al^[15]^ revealed that the distribution of distal LL (L4–S1) remains stable regardless of the variation in PI, while proximal lordosis increased with PI. Given that degenerative changes tend to occur primarily in the distal lumbar segment, spinal fusion procedures are frequently performed in this region.^[12]^ The restoration of the natural shape of regional lordosis is crucial in preventing the onset of ASD that commonly arises postoperatively. Against this backdrop, this investigation aimed to determine whether the creation of a specific angle during PLIF at the L4–5–S1 level has a significant impact on the incidence of postoperative ASD. We hypothesized that maintaining a stable distal lordotic angle independent of PI would be associated with a lower risk of postoperative ASD following L4–S1 PLIF.

## 2. Methods

A retrospective analysis was performed on a consecutive cohort of patients who underwent PLIF at the L4–5–S1 level for degenerative lumbar pathologies between January 2019 and December 2021, with a minimum follow-up period of 2 years. This retrospective study was approved by the institutional review board of our institution (IRB No. OC25RISI0172), and data used in the investigation were obtained from the electronic medical records of a single, tertiary academic referral center. Patients who met the inclusion criteria of severe low back pain characterized by radiculopathy or neurogenic claudication unresponsive to conservative treatment, and whose condition resulted from degenerative lumbar spinal stenosis or grade 1 or 2 spondylolisthesis, including spondylolysis, were included in the study. All PLIF surgeries in this study were performed by 3 experienced spine surgeons at the hospital where the authors were affiliated. The surgical technique involved a common procedure that consisted of bilateral L4 and L5 laminectomy followed by total discectomy at the L4–5 and L5–S1 levels with endplate preparation. Subsequently, the cage was inserted into the disc space by crumpling the resected lamina and filling it with demineralized bone matrix. Finally, bilateral L4, 5, and S1 pedicle screw fixations were performed. The exclusion criteria for this study were as follows: patients with a history of lumbar fusion or laminectomy surgery, those with spondylolisthesis grade 3 or higher, or scoliosis with a Cobb angle of 10° or more. Severe degeneration was also excluded, which was defined as a 50% reduction in disc height, a 3 mm slippage at L3–4, or a change in angulation of more than 5° on plain radiographs, as well as grade III or higher Pfirrmann degeneration on sagittal T2-weighted MR image at L3–4.^[16]^ Patients with tumors, infections, or trauma were also excluded from this study.

Patient demographic information such as age, sex, body mass index (BMI), and T-score were obtained from electronic medical records. Radiographic data were collected from whole-body standing lateral radiographs obtained before surgery, postoperatively, and at the 2-year follow-up. Radiographic evaluations included the assessment of the C7 sagittal vertical axis (SVA), LL, PI, sacral slope (SS), PT, and mismatch of PI and LL (PI–LL). The LL was segmented into 2 arcs: the upper arc, which spanned from the L1 superior endplate to the L4 superior endplate, and the lower arc, which extended from the L4 superior endplate to the S1 superior endplate. These arcs were referred to as proximal lordosis and distal lordosis, respectively, and the Cobb angles of each were quantified. LDI, represented by the equation distal lordosis/LL × 100%, serves to determine the degree of lower arc lordosis relative to overall global lordosis. The radiographic parameters were measured by 2 trained and independent observers using validated software (Surgimap, Nemaris Inc., New York), and the average of the 2 measurements was used for subsequent analysis.

The primary outcome measure was the development of ASD within the first 2 years postoperatively. ASD refers to segmental kyphosis of more than 10°, development of anterolisthesis or retrolisthesis of more than 3 mm, and deterioration in the Pfirrmann classification of one grade or greater progression at the level adjacent to a previous lumbar fusion.^[17]^ Secondary outcome measures included revision surgery due to clinical deterioration caused by ASD during the follow-up period. The cohorts were divided into 2 distinct groups based on the observed outcomes: one group consisted of subjects who developed ASD during the follow-up period (ASD group), and the other group consisted of subjects who did not (non-ASD group).

## 3. Statistical analysis

Descriptive statistics, such as mean and standard deviation, were used to summarize continuous variables, whereas count and percentage were used to describe categorical variables in this study. An independent two-sample *t*-test was used to compare continuous variables between groups, while the chi-square test or Fisher exact test was applied to compare categorical variables based on sample size. The normality of the variables was evaluated using the Kolmogorov–Smirnov test. A significance level of *P* < .05 was considered statistically significant.

To investigate the correlation between LL, proximal lordosis, distal lordosis, LDI, and PI, Pearson correlation or Spearman correlation analysis was performed in both groups, depending on the results of the normality tests. The patients were categorized into 3 subgroups based on their PI values: small PI (PI < mean −1 SD), average PI (PI mean ± 1 SD), and large PI (PI > mean + 1 SD). For each subgroup in both the ASD and non-ASD groups, lower lumbar lordosis and LDI were reported, and a comparison was made using ANOVA or the Kruskal–Wallis test. Post hoc comparisons were conducted using either the Scheffe test or Dunn multiple comparison test, depending on the specific analysis performed.

## 4. Results

**Figure 1.**
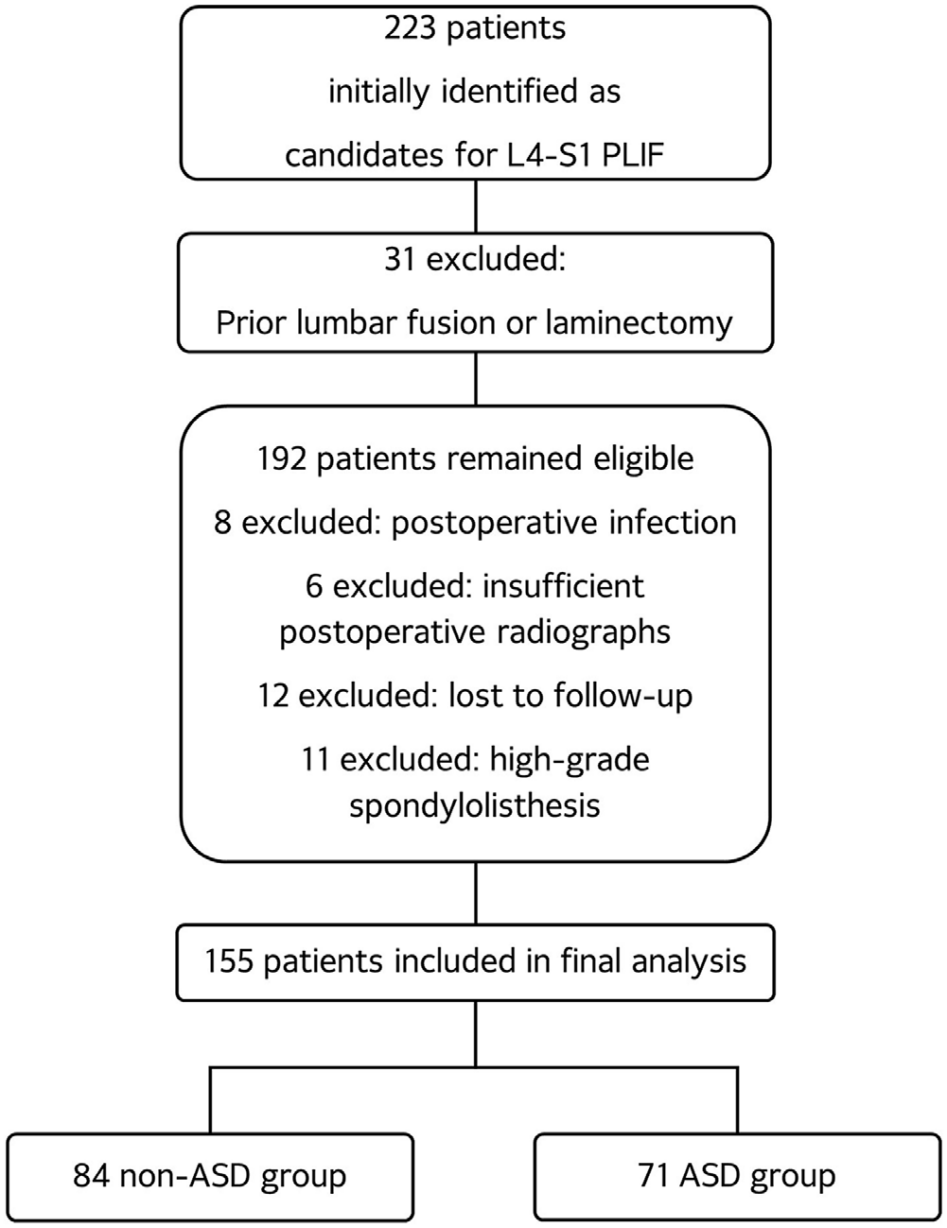
Flowchart of patient inclusion. ASD = adjacent segment degeneration, PLIF = posterior lumbar interbody fusion.

A total of 223 patients were initially identified as suitable candidates for L4–S1 PLIF surgery, of whom 31 were deemed ineligible due to prior fusion or laminectomy. Subsequently, 192 patients met the eligibility criteria; however, 8 were excluded due to postoperative infection, 6 had insufficient postoperative radiographs, and 12 were lost to follow-up. Furthermore, 11 patients who underwent PLIF for high-grade spondylolisthesis (grade 3 or higher) were excluded, resulting in a final cohort of 155 patients for analysis (Fig. 1). Following L4–S1 PLIF surgery, the final follow-up period encompassed 39.60 ± 20.33 months, during which 71 out of 155 patients (45.8%) were diagnosed with ASD. Of these, 12 (7.7%) required revision surgery. The non-ASD group consisted of 84 patients (39 male and 45 female) with a mean age of 64.28 ± 10.48 years. The average BMI at admission for this group was 24.69 ± 4.39 kg/m^2^, and the T score was −0.50 ± 1.90. The ASD group consisted of 71 patients (31 male and 40 female) with a mean age of 66.07 ± 6.07 years. The average BMI at admission for this group was 25.05 ± 3.37 kg/m^2^, and the T score was −1.19 ± 1.35. Regarding these demographic variables, no statistically significant differences were found between the 2 groups (Table 1).

**Table 1:** Comparison of preoperative baseline data from non-ASD and ASD group.

	Non-ASD group	ASD group	*P* value
No. of patients (%)	84	71	–
Age	64.28 ± 10.48	66.07 ± 6.07	.363
Gender (n, %)			
Female	49	40	.930
Male	35	31	
T-score	−0.50 ± 1.90	−1.19 ± 1.35	.102
BMI (kg/m^2^)	24.69 ± 4.39	25.05 ± 3.37	.700

ASD = adjacent segment degeneration, BMI = body mass index.

Table 2 presents the measurements of spinal parameters obtained from preoperative radiographs. The sagittal alignment of patients in the ASD group was comparatively poorer than that of their non-ASD counterparts, with similar values of PI (*P* = .092). The ASD group presented with a larger SVA (41.38 ± 25.10 mm vs 23.85 ± 19.89 mm, *P* = .019) and PT (25.81 ± 9.80° vs 21.17 ± 7.32°, *P* = .035) along with smaller SS (27.33 ± 9.88° vs 35.38 ± 7.98°, *P* < .001) and LL (31.28 ± 14.20° vs 41.25 ± 11.91°, *P* = .003). Furthermore, the ASD group exhibited a more pronounced PI–LL mismatch (21.86 ± 14.26° vs 15.30 ± 11.12°, *P* = .042) and a smaller angle at the L4–S1 level 23.21 ± 11.48° vs 29.49 ± 7.10°, *P* = .012). No significant differences in proximal lordosis and LDI were observed between the 2 groups. (*P* = .083 and 0.177, respectively).

**Table 2:** Comparison of preoperative radiographic parameters between the non-ASD and ASD group.

	Non-ASD group	ASD group	*P* value
Sagittal vertical axis (mm)	23.85 ± 19.89	41.38 ± 25.10	**.019**
Pelvic incidence (°)	56.55 ± 7.37	53.14 ± 8.63	.092
Pelvic tilt (°)	21.17 ± 7.32	25.81 ± 9.80	**.035**
Sacral slope (°)	35.38 ± 7.98	27.33 ± 9.88	**<.001**
Lumbar lordosis (°)	41.25 ± 11.91	31.28 ± 14.20	**.003**
Proximal lordosis (°)	11.76 ± 9.20	8.06 ± 8.40	.083
Distal lordosis (°)	29.49 ± 7.10	23.21 ± 11.48	**.012**
Lordosis distribution index (%)	75.49 ± 21.74	87.22 ± 64.78	.177
PI−LL (°)	15.30 ± 11.12	21.86 ± 14.26	**.042**

Bold text indicates statistically significant values at the *P* < .05 level.

ASD = adjacent segment degeneration, PI−LL = pelvic incidence minus lumbar lordosis.

Table 3 presents the postoperative measurements of spinal parameters, where patients with ASD exhibited greater PT (24.61 ± 8.28°) and smaller SS (27.70 ± 10.25°) and LL (32.84 ± 13.78°) than non-ASD patients (19.19 ± 6.39°; *P* = .002, 36.15 ± 7.96°; *P* < .001, 45.37 ± 11.62°; *P* < .001, respectively), despite having no statistically significant difference in SVA values (*P* = .141). Furthermore, the PI–LL mismatch was more pronounced in the ASD group (19.48 ± 11.56° vs 9.98 ± 10.07°, *P* < .001). Although there was no significant difference in LDI values between the 2 groups (*P* = .081), patients with ASD exhibited a smaller angle of distal lordosis (24.73 ± 10.86° vs 30.93 ± 6.97°, *P* = .005).

**Table 3:** Comparison of postoperative radiographic parameters between the non-ASD and ASD group.

	Non-MC group	MC group	*P*-value
Sagittal vertical axis (mm)	11.61 ± 19.55	18.41 ± 28.56	.141
Pelvic incidence (°)	55.35 ± 7.22	52.32 ± 8.99	.137
Pelvic tilt (°)	19.19 ± 6.39	24.61 ± 8.28	**.002**
Sacral slope (°)	36.15 ± 7.96	27.70 ± 10.25	**<.001**
Lumbar lordosis (°)	45.37 ± 11.62	32.84 ± 13.78	**<.001**
Proximal lordosis (°)	14.43 ± 8.33	8.10 ± 7.28	**.001**
Distal lordosis (°)	30.93 ± 6.97	24.73 ± 10.86	**.005**
Lordosis distribution index (%)	70.15 ± 14.58	78.68 ± 22.73	.081
PI−LL	9.98 ± 10.07	19.48 ± 11.56	**<.001**

Bold text indicates statistically significant values at the *P* < .05 level.

ASD = adjacent segment degeneration, PI−LL = pelvic incidence minus lumbar lordosis.

**Figure 2.**
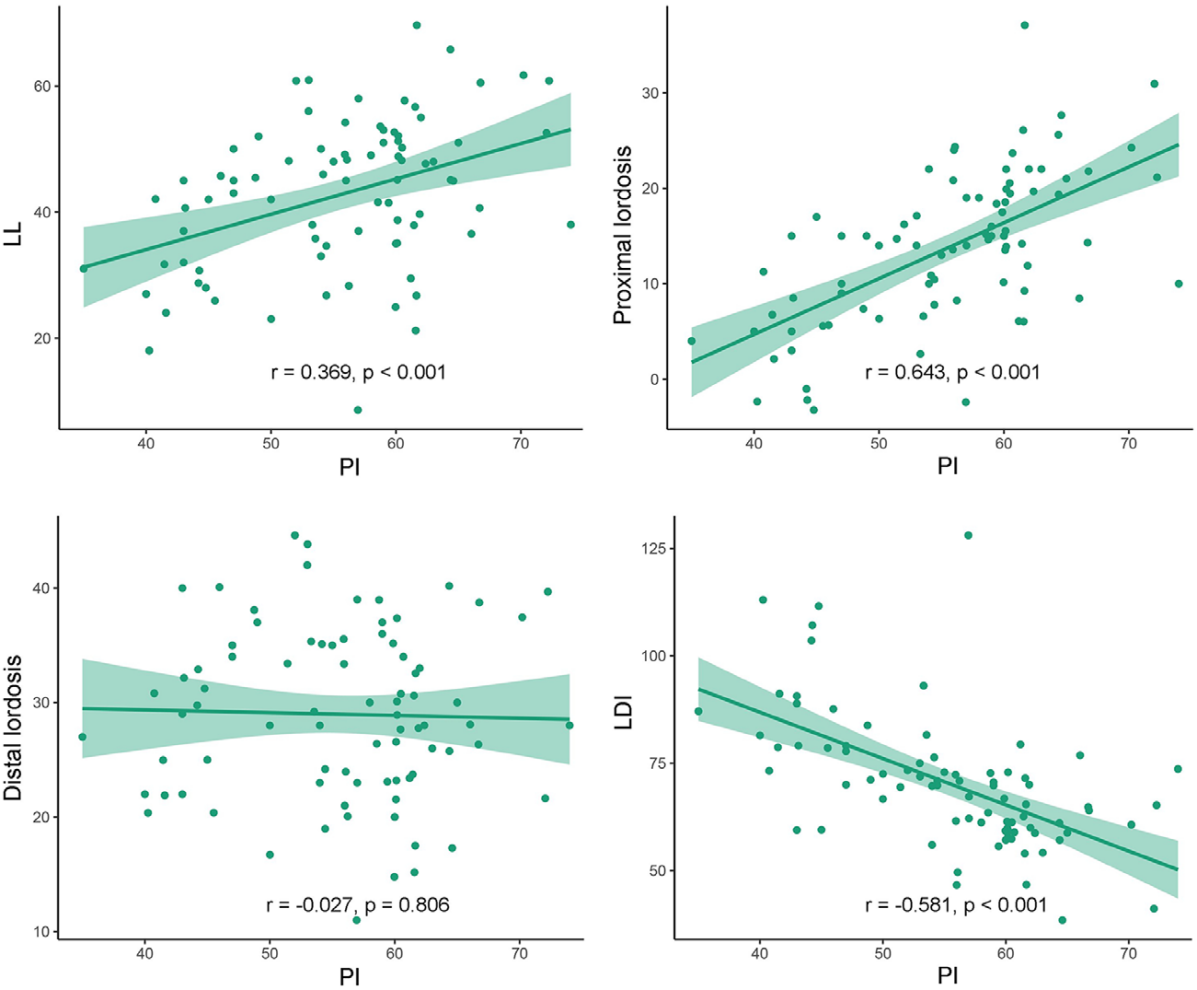
Linear regression between the PI and other parameters in the non-ASD group. ASD = adjacent segment degeneration, LDI = lordosis distribution index, LL = lumbar lordosis, PI = pelvic incidence.

**Figure 3.**
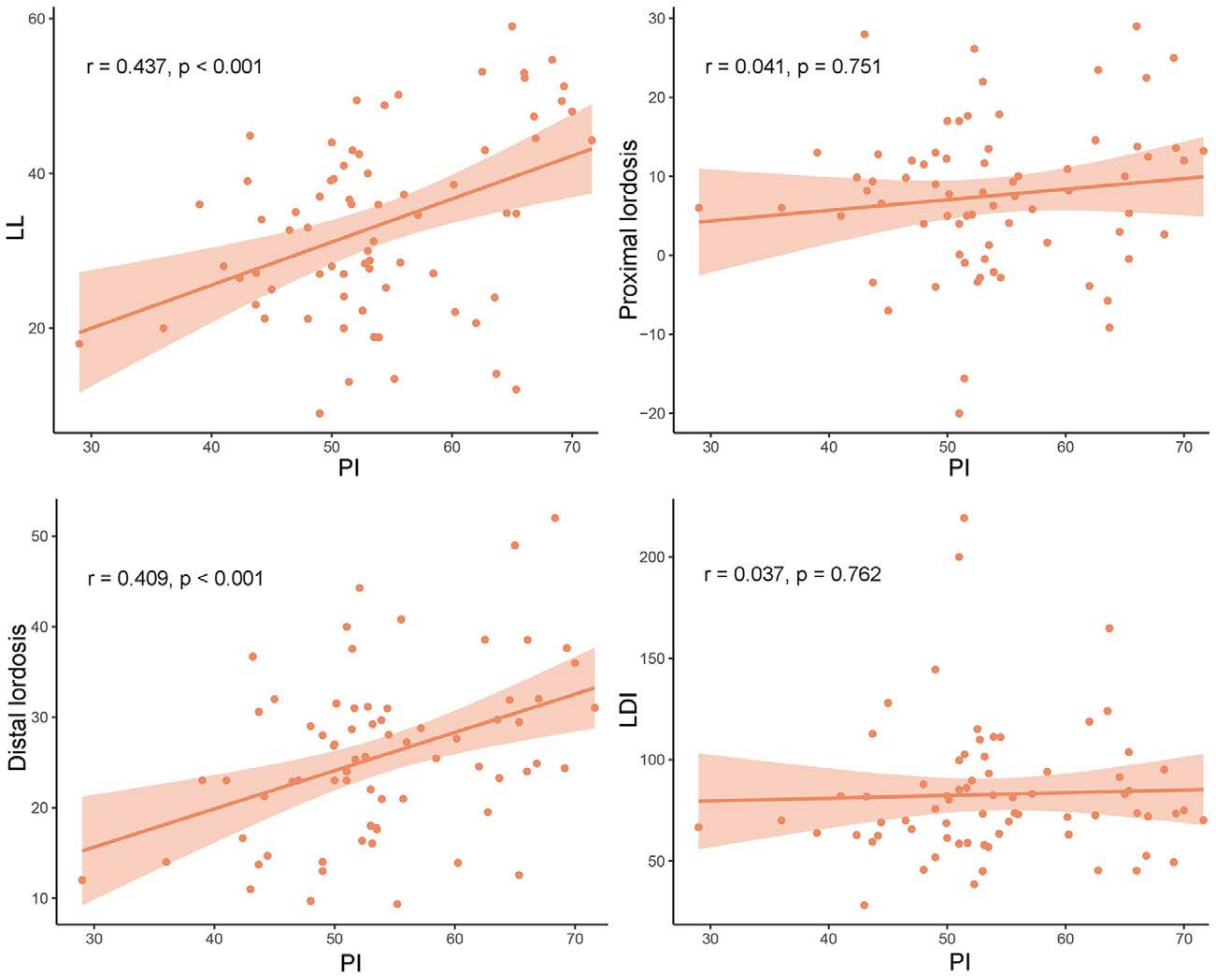
Linear regression between the PI and other parameters in the ASD group. ASD = adjacent segment degeneration, LDI = lordosis distribution index, LL = lumbar lordosis, PI = pelvic incidence.

A detailed depiction of the association between PI and other sagittal lumbar parameters in the non-ASD and ASD groups is presented in Figures 2 and 3, respectively. The PI was positively correlated with LL in both the non-ASD (*r* = 0.369, *P* < .001) and ASD groups (*r* = 0.437, *P* < .001). However, the correlations of PI with proximal lordosis, distal lordosis, and LDI differed between the 2 groups. Specifically, while distal lordosis exhibited a significant positive correlation with PI in the ASD group (*r* = 0.409, *P* < .001), it did not demonstrate a statistically significant correlation in the non-ASD group (*r* = −0.027, *P* = .806). Additionally, in the non-ASD group, an increase in PI was associated with an increase in proximal lordosis (*r* = 0.643, *P* < .001) and a decrease in LDI (*r* = −0.581, *P* < .001). Conversely, in the ASD group, PI was not significantly correlated with either proximal lordosis (*r* = 0.041, *P* = .751) or LDI (*r* = 0.037, *P* = .762).

**Figure 4.**
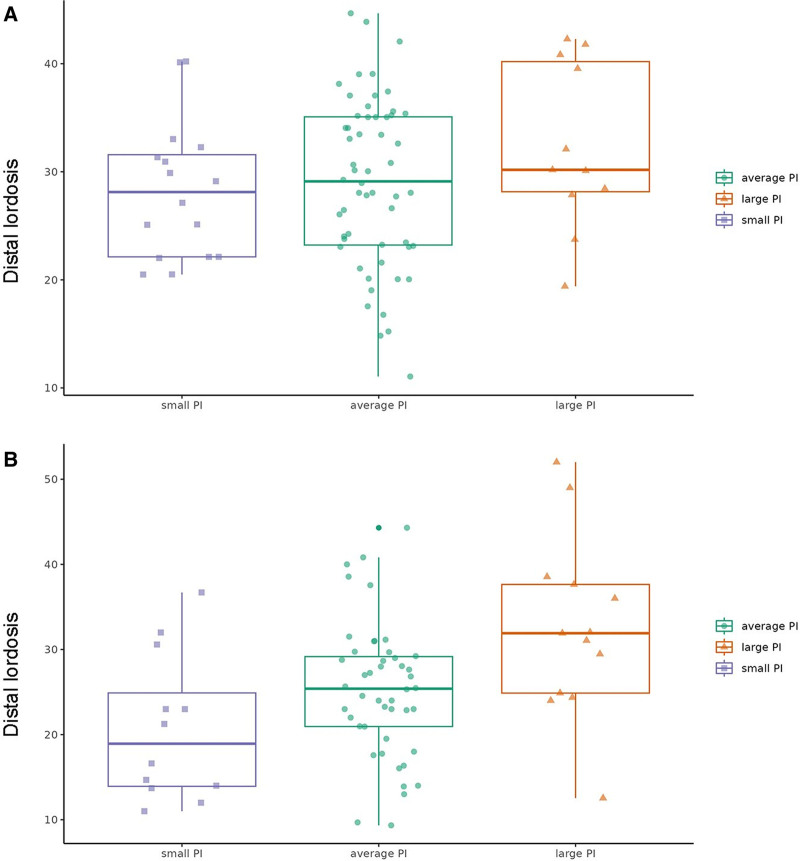
Box and jitter plots depict postoperative distal lordosis in non-ASD and ASD patient groups (A and B, respectively), stratified by small, average, and large PI categories. ASD = adjacent segment degeneration, PI = pelvic incidence.

Table 4 and Figure 4 compare the mean postoperative distal lordosis in patients with small, average, and large PI in both the non-ASD and ASD groups. The results were graphically presented, allowing easy visualization of the differences. The mean preoperative PI value in the current study was 55.00 ± 9,17° (minimum, 32.00°; maximum, 95.65°), showing normal distribution (Kolmogorov–Smirnov test, *P* = .825). In the non-ASD group, the mean distal lordosis did not show a statistically significant difference among the patients with different PI values (*P* = .758). In contrast, in the ASD group, distal lordosis showed statistically significant differences (*P* = .003) among PI subgroups. There was no statistically significant difference between the small and average PI groups (*P* = .324); however, the large PI group showed a statistically significant difference compared to both the small (*P* = .003) and average (*P* = .022) PI groups.

**Table 4:** Distal lordosis in patients with small, average, and large PI in non-ASD and ASD group.

Distal lordosis (°)	Small PI, < 46°	Average PI, 46°–64°	Large PI, > 64°	*P* value	Small vs Average *P* value	Average vs Large *P* value	Large vs Small *P* value
Non-ASD group	28.09 ± 6.31	28.97 ± 7.73	30.28 ± 7.73	.758	.918	.869	.758
ASD group	20.71 ± 8.57	25.19 ± 7.82	32.57 ± 10.54	**.003**	.324	**.022**	**.003**

Bold text indicates statistically significant values at the *P* < .05 level.

ASD = adjacent segment degeneration, PI = pelvic incidence.

## 5. Discussion

It has been documented that inferior preoperative spinopelvic alignment is associated with an increased probability of mechanical complications after lumbar fusion.^[10,18]^ The current study showed that while the mean values of PI were comparable between the non-ASD and ASD groups, the latter exhibited worse preoperative lumbopelvic sagittal parameters. Postoperatively, the ASD group showed a significant PI–LL mismatch of >10°, which may be related to insufficient restoration of LL. However, the inadequacy of the PI–LL classification system in providing proper guidance for achieving an optimal lumbar shape and adequate distribution during lumbar fusion surgery is a widely acknowledged issue in the field. Moreover, the system poses significant challenges in determining the angle and proportion of the proximal and distal lumbar segments as well as identifying the lumbar apex within its framework. This difficulty is further compounded when the procedure is performed on a short L4–S1 segment as opposed to the entire lumbar spine.^[19]^ This is primarily because of the influential role of the L4–S1 segment, which accounts for 62% of the total LL.^[15]^ The geometric characteristics of this segment are highly linked to SS, which has a stronger association with LL than PI.^[20]^ This association adds complexity to the task. Therefore, it is crucial to recognize that the equilibrium between the angles of the proximal and distal LL, as well as the location of the lumbar apex, can influence the distribution of load and the probability of degeneration in adjacent spinal segments.^[11,12]^

Yilgor et al^[14]^ incorporated LDI as a parameter within the global alignment and proportion score to forecast the incidence of mechanical complications after long-segment lumbar fusion. They argued that LDI values below 50% are indicative of hypolordotic malalignment, whereas those above 80% indicate hyperlordotic malalignment. These deviations increase the risk of mechanical complications. Bari et al^[11]^ observed an augmented revision rate in patients with an LDI of 50% or less who underwent short-level lumbar fusion, while Zheng et al^[12]^ reported that, after a monitoring period of 84 months, 200 patients who underwent L4–S1 PLIF had a postoperative ASD rate of 25% in the low LDI group and 18.4% in the high LDI group, both significantly higher than the 4.1% incidence rate in the aligned LDI group (*P* = .006 and .007, respectively). Additionally, low LDI was more likely to result in ASD than high LDI. However, the current investigation yielded disparate outcomes when compared to previous publications, given that the postoperative LDI values of both cohorts ranged between 50% and 80%, which denotes a state of alignment, and no statistically significant difference was detected between the groups. Tobert et al^[21]^ have cast doubt on the accuracy of the LDI classification system, as they discovered that when treated as a continuous variable, LDI was significantly linked to mechanical failure, whereas there was no significant correlation when treated as a categorical variable. This implies that defining the physiological and pathological limits of LDI requires meticulous deliberation. In addition, we believe that the significance of LDI in the context of ASD development may have been underestimated due to the failure of previous literature to consider the interplay between LDI categorization and PI.

Roussouly et al^[13]^ proposed that the shape of LL can be categorized into 4 distinct types within the asymptomatic adult population, based on the orientation of SS and the size of PI. Each type has distinct spinopelvic morphological characteristics. Their research findings revealed that individuals with low PI tend to have relatively flat and short lordosis, with a relatively larger proportion of the lower lumbar portion comprising total lordosis. The smallest PI type, Type 1, exhibited an LDI value as high as 90%. In contrast, individuals with a larger PI display curved and long lordosis, with type 4 demonstrating an LDI value of 60%. The current study discovered that in the non-ASD group, there was a decline in LDI value as PI value increased, while there was no substantial correlation between these parameters in the ASD group. The reason for the negative correlation is likely due to the fact that proximal lordosis increases with PI, while the distal L4–S1 portion remains consistent across different PI values in the non-ASD group. Pesenti et al^[15]^ conducted a study involving 119 asymptomatic volunteers, which revealed a positive correlation (*r* = 0.546; *P* < .001) between PI and proximal lordosis, whereas distal lordosis remained relatively constant and uninfluenced by PI (*r* = 0.087; *P* = .346). Based on these findings, they deduced that as PI increased, proximal levels were called upon to enhance lordosis, whereas distal lordosis did not appear to contribute to the overall shape of lordosis. Hills et al^[22]^ conducted a study on sagittal alignment among healthy individuals from 5 different countries and observed that distal lordosis exhibited a nonconstant pattern but did not exhibit any substantial correlation with PI. In a follow-up study on the relationship between proximal and distal lordosis and PI, Pesenti et al^[23]^ found that deficient restoration of the L4–S1 segment following lumbar fusion was associated with an increased PI–LL mismatch, as well as unfavorable outcomes regarding ASD and clinical prognosis.

Attaining sufficient restoration of the L4–S1 segment is a crucial objective in lumbar fusion surgery, as this region is prone to degenerative changes and its effect on sagittal alignment. Lafage et al^[24]^ have reported that 1° correction in the L4–S1 angle can lead to a significant 10 mm alteration in SVA and 0.5° modification in PT. Furthermore, caudal kyphotic changes can result in proximal junctional failure when a greater cranial lordotic correction is made to achieve the desired total LL correction.^[25]^ Therefore, effective management strategies that optimize local lordosis may help reduce the risk of ASD, as the distal lordosis in the ASD group was significantly smaller than in the non-ASD group in this study. However, the establishment of an appropriate angle at the L4–S1 level is a subject of deliberation among the spine community and researchers, primarily because of the paucity of research and limited evidence available on this topic. Pesenti et al^[15]^ classified the cohort into 3 categories based on PI: a low PI group (PI ≤ 45°), an average PI group (PI between 45° and 60°), and a high PI group (PI ≥ 60°). They then conducted a comparative analysis of distal lordosis among these groups and found no significant differences in distal lordosis across the different PI groups. Additionally, the study found no association between distal lordosis and PI, with an angle of approximately 35° in all the groups. The current study found that the mean postoperative distal lordosis in the non-ASD group was similar across all the PI-stratified groups (approximately 30°). This result is consistent with the findings of previous studies. Zheng et al^[12]^ reported a mean postoperative lower lumbar lordosis of 28.82°, while Pesenti et al^[23]^ observed a value of 31°. Based on our observations, maintaining a distal lordosis of approximately 30° may be associated with a lower likelihood of ASD. However, it should be noted that this value was lower than the previously suggested value of 35°. The average age of the current study’s cohort was in their 60s, which is older than Pesenti et al^[15]^ cohort who were in their 50s. Therefore, when determining alignment goals, it is important to take into consideration age-adjusted alignment targets that are associated with degenerative changes.^[26]^ Additionally, ethnic differences should also be considered, as Asians tend to have less lordotic lumbar curvature than Westerners who are more likely to have Roussouly type 2 than type 3.^[27]^

The current investigation had several limitations. First, the sample size was limited, and a larger cohort would allow for a more comprehensive examination of the association between pelvic morphology and lumbar shape. Second, while it would be beneficial to explore the effects of additional PI groups as well as the influence of demographic factors such as age, sex, and ethnicity, this would necessitate a larger study population. Third, the development of ASD is multifactorial in nature. Unfortunately, the present study did not account for the impact of the distal lordosis angle, nor did it evaluate the odds ratios for the various risk factors contributing to ASD following L4–S1 PLIF. These associations should be interpreted cautiously, as potential confounding factors cannot be excluded in this retrospective study. Lastly, the implications of the postoperative L4–S1 angle on clinical outcomes were not analyzed. Nevertheless, previous research^[11,24]^ has shown that ASD is associated with a greater likelihood of revision surgery and poorer prognosis. Therefore, we recommend that future prospective investigations with larger participant cohorts be undertaken to verify these findings.

## 6. Conclusions

In addition to the restoration of proper LL in line with pelvic morphology, the reestablishment of harmony between regional lumbar curvatures, which should be consistent with the natural shape of a patient’s spine, is crucial to avoid unfavorable outcomes. Despite a wide variation in proximal lordosis, distal lordosis was withing a narrower range, which showed an independent relationship with PI in patients without ASD following L4–S1 PLIF. As a result, surgeons should consider analyzing the distal lordotic angle to restore an appropriate LL distribution, which may contribute to reducing the risk of ASD.

## Author contributions

**Conceptualization:** Myung-Hoon Shin.

**Formal analysis:** Young-Gook Gwak, Myung-Hoon Shin.

**Investigation:** Young-Gook Gwak.

**Methodology:** Myung-Hoon Shin.

**Supervision:** Myung-Hoon Shin.

**Writing – original draft:** Myung-Hoon Shin.

**Writing – review & editing:** Myung-Hoon Shin.

## References

[1] Cloward RB. The treatment of ruptured lumbar intervertebral discs by vertebral body fusion. I. Indications, operative technique, after care. J Neurosurg. 1953;10:154–68. 13035484 10.3171/jns.1953.10.2.0154

[2] Hutter CG. Posterior intervertebral body fusion A 25-year study. Clin Orthop Relat Res. 1983;179:86–96. 6352135

[3] Simeone FA Garfin SR Eismont FJ. Rothman-Simeone and Herkowitz’s The Spine. 2 vol set. Elsevier; 2017.

[4] Jiang S Li W. Biomechanical study of proximal adjacent segment degeneration after posterior lumbar interbody fusion and fixation: a finite element analysis. J Orthop Surg Res. 2019;14:135. 31092257 10.1186/s13018-019-1150-9 PMC6521416

[5] Lawrence BD Wang J Arnold PM Hermsmeyer J Norvell DC Brodke DS. Predicting the risk of adjacent segment pathology after lumbar fusion: a systematic review. Spine (Phila Pa 1976). 2012;37:S123–32. 22885827 10.1097/BRS.0b013e31826d60d8

[6] Legaye J Duval-Beaupère G Hecquet J Marty C. Pelvic incidence: a fundamental pelvic parameter for three-dimensional regulation of spinal sagittal curves. Eur Spine J. 1998;7:99–103. 9629932 10.1007/s005860050038 PMC3611230

[7] Smith JS Klineberg E Schwab F. Change in classification grade by the SRS-Schwab Adult spinal deformity classification predicts impact on health-related quality of life measures: prospective analysis of operative and nonoperative treatment. Spine (Phila Pa 1976). 2013;38:1663–71. 23759814 10.1097/BRS.0b013e31829ec563

[8] Kyrölä K Repo J Mecklin JP Ylinen J Kautiainen H Häkkinen A. Spinopelvic changes based on the simplified SRS-Schwab adult spinal deformity classification: relationships with disability and health-related quality of life in adult patients with prolonged degenerative spinal disorders. Spine (Phila Pa 1976). 2018;43:497–502. 28767623 10.1097/BRS.0000000000002370

[9] Phan K Nazareth A Hussain AK. Relationship between sagittal balance and adjacent segment disease in surgical treatment of degenerative lumbar spine disease: meta-analysis and implications for choice of fusion technique. Eur Spine J. 2018;27:1981–91. 29808425 10.1007/s00586-018-5629-6

[10] Soroceanu A Diebo BG Burton D. Radiographical and implant-related complications in adult spinal deformity surgery: incidence, patient risk factors, and impact on health-related quality of life. Spine (Phila Pa 1976). 2015;40:1414–21. 26426712 10.1097/BRS.0000000000001020

[11] Bari TJ Heegaard M Bech-Azeddine R Dahl B Gehrchen M. Lordosis distribution index in short-segment lumbar spine fusion – can ideal lordosis reduce revision surgery and iatrogenic deformity? Neurospine. 2021;18:543–53. 34610685 10.14245/ns.2040744.372 PMC8497240

[12] Zheng G Wang C Wang T. Relationship between postoperative lordosis distribution index and adjacent segment disease following L4-S1 posterior lumbar interbody fusion. J Orthop Surg Res. 2020;15:129. 32245387 10.1186/s13018-020-01630-9 PMC7119009

[13] Roussouly P Gollogly S Berthonnaud E Dimnet J. Classification of the normal variation in the sagittal alignment of the human lumbar spine and pelvis in the standing position. Spine (Phila Pa 1976). 2005;30:346–53. 15682018 10.1097/01.brs.0000152379.54463.65

[14] Yilgor C Sogunmez N Boissiere L. Global alignment and proportion (GAP) score: development and validation of a new method of analyzing spinopelvic alignment to predict mechanical complications after adult spinal deformity surgery. J Bone Joint Surg Am. 2017;99:1661–72. 28976431 10.2106/JBJS.16.01594

[15] Pesenti S Lafage R Stein D. The amount of proximal lumbar lordosis is related to pelvic incidence. Clin Orthop Relat Res. 2018;476:1603–11. 29965893 10.1097/CORR.0000000000000380 PMC6259763

[16] Duan PG Mummaneni PV Berven SH. Revision surgery for adjacent segment degeneration after fusion for lumbar spondylolisthesis: is there a correlation with Roussouly type? Spine (Phila Pa 1976). 2022;47:E10–5. 32991517 10.1097/BRS.0000000000003708

[17] Cheh G Bridwell KH Lenke LG. Adjacent segment disease following lumbar/thoracolumbar fusion with pedicle screw instrumentation: a minimum 5-year follow-up. Spine (Phila Pa 1976). 2007;32:2253–7. 17873819 10.1097/BRS.0b013e31814b2d8e

[18] Kim HJ Bridwell KH Lenke LG. Patients with proximal junctional kyphosis requiring revision surgery have higher postoperative lumbar lordosis and larger sagittal balance corrections. Spine (Phila Pa 1976). 2014;39:E576–80. 24480958 10.1097/BRS.0000000000000246

[19] Ge T Xie L Li J Ao J Wu J Sun Y. Lumbar lordosis distribution in asymptomatic adult volunteers: a systematic review. HSS J. 2023;19:223–33. 37065105 10.1177/15563316221145156 PMC10090846

[20] Schwab F Lafage V Patel A Farcy J-P. Sagittal plane considerations and the pelvis in the adult patient. Spine (Phila Pa 1976). 2009;34:1828–33. 19644334 10.1097/BRS.0b013e3181a13c08

[21] Tobert DG Davis BJ Annis P. The impact of the lordosis distribution index on failure after surgical treatment of adult spinal deformity. Spine J. 2020;20:1261–6. 32200117 10.1016/j.spinee.2020.03.010

[22] Hills J Lenke LG Sardar ZM. The T4-L1-hip axis: defining a normal sagittal spinal alignment. Spine (Phila Pa 1976). 2022;47:1399–406. 35867583 10.1097/BRS.0000000000004414

[23] Pesenti S Prost S McCausland AM. Optimal correction of adult spinal deformities requires restoration of distal lumbar lordosis. Adv Orthop. 2021;2021:5572181. 34040810 10.1155/2021/5572181 PMC8121594

[24] Lafage R Obeid I Liabaud B. Location of correction within the lumbar spine impacts acute adjacent-segment kyphosis. J Neurosurg Spine. 2018;30:69–77. 30485215 10.3171/2018.6.SPINE161468

[25] Lafage R Schwab F Elysee J. Surgical planning for adult spinal deformity: anticipated sagittal alignment corrections according to the surgical level. Global Spine J. 2022;12:1761–9. 33567927 10.1177/2192568220988504 PMC9609531

[26] Lafage R Schwab F Glassman S. Age-adjusted alignment goals have the potential to reduce PJK. Spine (Phila Pa 1976). 2017;42:1275–82. 28263226 10.1097/BRS.0000000000002146

[27] Ma H Hu Z Shi B. Global alignment and proportion (GAP) score in asymptomatic individuals: is it universal? Spine J. 2022;22:1566–75. 35447324 10.1016/j.spinee.2022.04.003

